# Study on Local-Structure Symmetrization of K_2_XF_6_ Crystals Doped with Mn^4+^ Ions by First-Principles Calculations

**DOI:** 10.3390/ma16114046

**Published:** 2023-05-29

**Authors:** Mega Novita, Sigit Ristanto, Ernawati Saptaningrum, Slamet Supriyadi, Dian Marlina, Ferdy Semuel Rondonuwu, Alok Singh Chauhan, Benjamin Walker, Kazuyoshi Ogasawara, Michal Piasecki, Mikhail G. Brik

**Affiliations:** 1 Postgraduate Program of Science Education, Universitas PGRI Semarang, Semarang 50232, Indonesia; 2 Faculty of Mathematics, Natural Sciences and Information Technology Education, Universitas PGRI Semarang, Semarang 50232, Indonesia; sigitristanto@upgris.ac.id (S.R.); ernawati@upgris.ac.id (E.S.); 3 Faculty of Engineering and Informatics, Universitas PGRI Semarang, Semarang 50232, Indonesia; slametsupriyadi@upgris.ac.id; 4 Faculty of Pharmacy, Universitas Setia Budi, Surakarta 57127, Indonesia; marlina@setiabudi.ac.id; 5 Department of Physics, Universitas Kristen Satya Wacana, Salatiga 50711, Indonesia; ferdy.rondonuwu@uksw.edu; 6 Department of Computer Application, Galgotias University, Greater Noida 203201, India; alok.chauhan@galgotiasuniversity.edu.in; 7 Independent Researcher, 925 Dalney Street NW, Atlanta, GA 30318, USA; benjamin.walker@gtri.gatech.edu; 8 School of Science and Technology, Kwansei Gakuin University, Sanda 669-1337, Japan; ogasawara@kwansei.ac.jp; 9 Department of Theoretical Physics, Jan Dlugosz University, 42-200 Czestochowa, Poland; m.piasecki@ujd.edu.pl (M.P.); m.brik@ujd.edu.pl (M.G.B.); 10 Centre of Excellence for Photoconversion, Vinča Institute of Nuclear Sciences-National Institute of the Republic of Serbia, University of Belgrade, 11351 Belgrade, Serbia; 11 School of Optoelectronic Engineering and CQUPT-BUL Innovation Institute, Chongqing University of Posts and Telecommunications, Chongqing 400065, China; 12 Academy of Romanian Scientists, Ilfov Str. No. 3, 010071 Bucharest, Romania; 13 Institute of Physics, University of Tartu, W. Ostwald Str. 1, 50411 Tartu, Estonia; 14 Institute of Solid State Physics, University of Latvia, Kengaraga 8, LV-1063 Riga, Latvia

**Keywords:** A_2_BF_6_, Mn^4+^, hexafluorometallate, fluorides, luminescence, phosphor

## Abstract

The crystals of Mn^4+^-activated fluorides, such as those of the hexafluorometallate family, are widely known for their luminescence properties. The most commonly reported red phosphors are A_2_XF_6_: Mn^4+^ and BXF_6_: Mn^4+^ fluorides, where A represents alkali metal ions such as Li, Na, K, Rb, Cs; X=Ti, Si, Ge, Zr, Sn, B = Ba and Zn; and X = Si, Ge, Zr, Sn, and Ti. Their performance is heavily influenced by the local structure around dopant ions. Many well-known research organizations have focused their attention on this area in recent years. However, there has been no report on the effect of local structural symmetrization on the luminescence properties of red phosphors. The purpose of this research was to investigate the effect of local structural symmetrization on the polytypes of K_2_XF_6_ crystals, namely *O_h_*-K_2_MnF_6_, *C*_3*v*_-K_2_MnF_6_, *O_h_*-K_2_SiF_6_, *C*_3*v*_-K_2_SiF_6_, *D*_3*d*_-K_2_GeF_6_, and *C*_3*v*_-K_2_GeF_6_. These crystal formations yielded seven-atom model clusters. Discrete Variational Xα (DV-Xα) and Discrete Variational Multi Electron (DVME) were the first principles methods used to compute the Molecular orbital energies, multiplet energy levels, and Coulomb integrals of these compounds. The multiplet energies of Mn^4+^ doped K_2_XF_6_ crystals were qualitatively reproduced by taking lattice relaxation, Configuration Dependent Correction (CDC), and Correlation Correction (CC) into account. The ^4^*A*_2*g*_→^4^*T*_2*g*_ (^4^F) and ^4^*A*_2*g*_→^4^*T*_1*g*_ (^4^F) energies increased when the Mn-F bond length decreased, but the ^2^*E_g_* → ^4^*A*_2*g*_ energy decreased. Because of the low symmetry, the magnitude of the Coulomb integral became smaller. As a result, the decreasing trend in the R-line energy could be attributed to a decreased electron–electron repulsion.

## 1. Introduction

Incandescent and fluorescent lighting sources have been rapidly replaced by White Light Emitting Diodes (WLEDs) in homes, offices, and public areas. They are made of a blue LED chip with a yellow phosphor. WLED is the most energy-efficient conversion source compared to previously existing lighting sources, yet it creates pseudo-white light due to a lack of red emissions. Rather than employing all basic colors of LED chips, mixing blue LED chips with yellow and red phosphors is simpler and less expensive. The blue LED chips are typically made of InGaN [1], whereas the yellow phosphor components are composed of Y_3_Al_5_O_12_: Ce^3+^ [2]. The high-performance red phosphors are Eu^2+^ doped nitrides [3,4,5,6,7,8,9]. Unfortunately, red phosphors are expensive due to scarcity and challenging synthesis conditions, such as extreme temperatures and nitrogen pressure. Finding novel red phosphor materials that are appropriate for WLED is currently challenging. Significant performance factors for white light that are used in general lighting include high Quantum Efficiency (QE > 70%), resistance to thermal quenching (preferably > 80% of the luminescence intensity should be sustained at 450 K), and strong color quality, which includes a low Correlated Color Temperature (CCT) of 3000 K and a high Color Rendering Index (CRI > 70).

The most commonly reported red phosphors are fluoride-based, such as A_2_XF_6_: Mn^4+^ and BXF_6_: Mn^4+^, where A represents alkali metal ions, such as Li, Na, K, Rb, Cs; X = Ti, Si, Ge, Zr, Sn), B = Ba and Zn and X = Si, Ge, Zr, Sn, and Ti. K_2_SiF_6_: Mn^4+^, KNa_2_SiF_6_: Mn^4+^, and K_2_TiF_6_: Mn^4+^, in particular, have shown good potential for WLED as a red phosphor under blue LED chip stimulation. The first red Mn^4+^-doped fluoride phosphor, K_2_SiF_6_: Mn^4+^, was published in 1973 [10]. K_2_SiF_6_ is one of the most promising hexafluoride hosts, with a slightly higher Luminous Efficacy of Radiation (LER) upon Mn^4+^ doping than K_2_TiF_6_ and a 30% higher External Quantum Efficiency (EQE) than KnaSiF_6_: Mn^4+^ [11]. Mn^4+^, when doped in K_2_SiF_6_ or K_2_TiF_6_ as a red phosphor, yields WLEDs with warm-white CCTs ~3000 K and good CRIs ~90, as demonstrated by Setlur et al. [12]. The d–d transitions in Mn^4+^ cause the particular red emission line detected in K_2_SiF_6_: Mn^4+^ to be approximately 630 nm (15,873 cm^−1^ or 1.97 eV) [13]. Nevertheless, the chemical and thermal stability problems and safety hazards of K_2_SiF_6_ and K_2_TiF_6_ doped with Mn^4+^ have been reported.

The aforementioned red-phosphor performance is highly dependent on local structure. Numerous research teams have concentrated on the modification and enhancement of phosphor luminescence properties through the alteration of the local crystal structure. The “Cation-Size-Mismatch effect”, “Neighboring-Cation Substitution effect”, and “Nanosegregation and Neighbor-Cation Control effect”, among other new luminescence mechanisms, were reported by Liu’s group in Ce^3+^ and Eu^2+^-doped (oxy)nitrides based on the variation in the local crystal structure [14,15,16,17]. Ram’s team also showed that slight modifications to the local structure of phosphor systems such as La_3_xCexSi_6_N_11_, SrxBa_2_xSiO_4_: Eu^2+^, etc., could lead to appreciable gains in luminescence performance [18,19]. Cheetham’s team discovered that local crystal structural deformation accounted for a significant spectrum change from blue to yellow light from Ca_2_SiO_4_: Ce [20].

The Ligand Field Theory (LFT) has been frequently used to successfully evaluate the multiplet energy levels and optical spectra of Transition Metal (TM) ions in crystals [21]. However, it is an empirical method in which the measured spectrum is used to determine the Racah parameters and crystal field splitting. Watanabe and Kamimura produced the first non-empirical forecast in the late 1980s [22,23] using a combination of the local density approximation (LDA) and LFT. On the other hand, a number of teams, including Daul et al. [24], Wissing et al. [25], and Oliveira et al. [26,27], have also performed first-principle calculations based on the Density Functional Theory (DFT). However, obtaining the many-electron wave functions proved unfeasible. During the previous ten years, Ogasawara’s team created the Discrete Variational Multi-Electron (DVME) approach [28]: a non-empirical first-principles many-electron calculation technique. It uses both a Configuration Interaction (CI) computation and DFT. DVME consists of two phases. To begin, one-electron Molecular Orbital (MO) calculations are performed using the Discrete Variational Xα (DV-Xα) method. The CI method is then used to perform many-electron computations, which is the main stage of the DVME approach. It has been shown that DVME is a powerful tool for estimating absorption spectra, energy levels, transition energies, etc., without the use of any empirical parameters.

Up until recently, there has been no study on the influence of local structure on symmetry (switching from a high-symmetry to a low-symmetry configuration) or on the luminous qualities of red phosphors. Therefore, the goal of this research was to investigate the effect of local structural symmetrization on the polytypes of K_2_XF_6_ crystals, namely *O_h_*−-K_2_MnF_6_, *C*_3*v*_-K_2_MnF_6_, *O_h_*-K_2_SiF_6_, *C*_3*v*_-K_2_SiF_6_, *D*_3*d*_-K_2_GeF_6_, and *C*_3*v*_-K_2_GeF_6_. The DVME method was used to calculate their multiplet energy levels.

## 2. Materials and Methods

**Figure 1:**
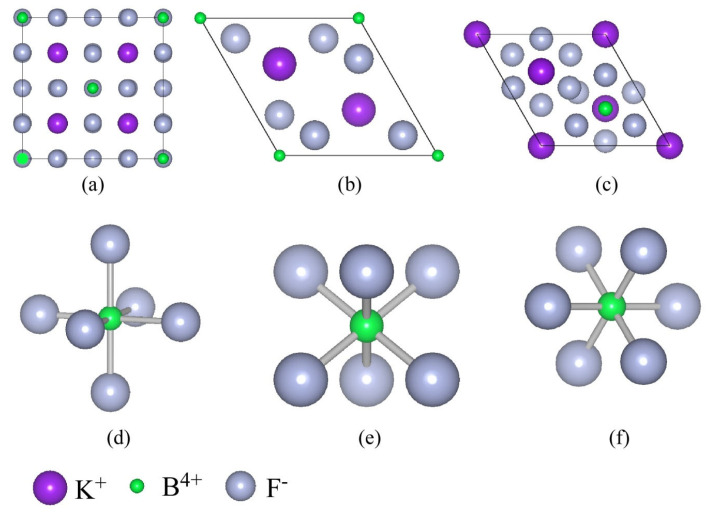
The crystal structure of K_2_XF_6_ (X = Mn, Si, or Ge) with (**a**) Cubic structure and space group $Fm\bar{3}m$, (**b**) Rhombohedral structure with space group $P\bar{3}m1$, and (**c**) Hexagonal structure with space group P63mc as seen from the *c* axis. The seven atoms represent clusters with (**d**) *O_h_*, (**e**) *D*_3*d*_, and (**f**) *C*_3*v*_ symmetry, with the Mn^4+^ ion in the core.

Polytypes of various K_2_XF_6_ crystals were used to create seven-atom model clusters. The cubic K_2_MnF_6_ ICSD #47213 had *a* = 8.221 lattice parameter, a space group $Fm\bar{3}m$, and *O_h_* symmetry [29]. The lattice parameters of hexagonal K_2_MnF_6_ ICSD #60417 were *a* = 5.719 Å and *c* = 9.330 Å, with space group P63mc and *C*_3*v*_ Symmetry [30]. The cubic K_2_SiF_6_ ICSD #2940 had *a*= 8.134 lattice parameters, a space group $Fm\bar{3}m$, and *O_h_* symmetry [31]. The lattice parameters of hexagonal K_2_SiF_6_ ICSD #158483 were *a* = 5.6461 Å and *c* = 9.2322 Å, with the space group P63mc and *C*_3*v*_ Symmetry [32]. The lattice parameters of rhombohedral K_2_GeF_6_ ICSD #24026 were *a* = 5.63 and *c* = 4.66, with the space group $P\bar{3}m1$ and *D*_3*d*_ Symmetry [33]. The lattice parameters of hexagonal K_2_GeF_6_ ICSD #30310 were *a* = 5.71 Å and *c* = 9.27 Å, with the space group P63mc and *C*_3*v*_ Symmetry [34]. The computations were performed using *O_h_*, *D*_3*d*_, and *C*_3*v*_ symmetry for clusters built from K_2_XF_6_ (X = Mn, Si, or Ge) and crystals with cubic, rhombohedral, and hexagonal structures, respectively. Figure 1a–c depicts the various types of crystal structures of the materials under consideration, including namely cubic, rhombohedral, and hexagonal structures. Figure 1d–f were model clusters made up of seven atoms, one X^4+^ ion surrounded by 6 F^−^. Here, we adopted the results of the Mn K-edge Extended X-ray Absorption Fine Structure (EXAFS) measurement of K_2_XF_6_ (X = Si, or Ge): Mn^4+^ [35]. The Mn-F bond lengths for K_2_SiF_6_: Mn^4+^ and K_2_GeF_6_: Mn^4+^ were 1.807 and 1.810 Å, respectively. The one-electron calculations utilizing the DV-Xα method were then carried out [36,37,38]. The DVME approach was used to account for the many-electron effects [28]. The energy corrections such as Configuration Dependent Correction (CDC) and Correlation Correction (CC) were also considered. Racah parameters were used to calculate the Coulomb integrals as well. These methods’ specific steps are described in Reference [35].

## 3. Results

### 3.1. Bond Lengths

The Mn-F bond lengths of *O_h_*-K_2_MnF_6_, *C*_3*v*_-K_2_MnF_6_, *O_h_*-K_2_SiF_6_, *C*_3*v*_-K_2_SiF_6_, *D*_3*d*_-K_2_GeF_6_, and *C*_3*v*_-K_2_GeF_6_. are shown in Table 1. All six bond lengths are represented by letters *d*1, *d*2, *d*3, *d*4, *d*5, and *d*6, respectively. When the lattice relaxation effect was not used, the lengths of the Mn-F bonds dropped from *O_h_*-K_2_MnF_6_ to *C*_3*v*_-K_2_MnF_6_. This was similar to the trend for *O_h_*-K_2_SiF_6_: Mn^4+^ to *C*_3*v*_-K_2_SiF_6_: Mn^4+^. On the other hand, the trend for *D*_3*d*_-K_2_GeF_6_: Mn^4+^ to *C*_3*v*_-K_2_GeF_6_: Mn^4+^ was reversed. When the lattice relaxation effect was used, however, the Mn-F bond lengths decreased in all situations.

### 3.2. Molecular Orbital Energies

**Figure 2:**
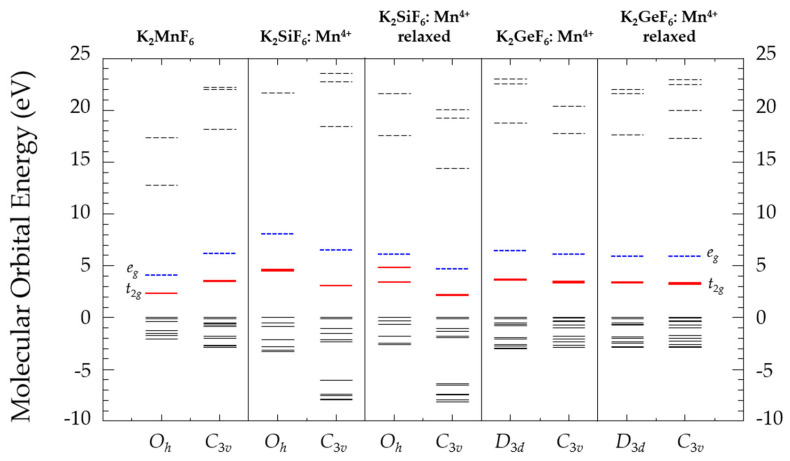
Molecular orbital energies of *O_h_*-K_2_MnF_6_, *C*_3*v*_-K_2_MnF_6_, *O_h_*-K_2_SiF_6_, *C*_3*v*_-K_2_SiF_6_, *D*_3*d*_-K_2_GeF_6_, and *C*_3*v*_-K_2_GeF_6_ doped with Mn^4+^. The Valence Band (VB) is represented by black solid lines. The Conduction Band (CB) is shown by the black dashed lines. The *t*_2*g*_ levels are indicated by the solid red lines, while the *e_g_* levels are indicated by the dashed blue lines.

Figure 2 depicts the molecular orbital energies of *O_h_*-K_2_MnF_6_, *C*_3*v*_-K_2_MnF_6_, *O_h_*-K_2_SiF_6_, *C*_3*v*_-K_2_SiF_6_, *D*_3*d*_-K_2_GeF_6_, and *C*_3*v*_-K_2_GeF_6_. The Valence Band (VB) is represented by black solid lines. The Conduction Band (CB) is shown by the black dashed lines. The impurity levels are represented as *t*_2*g*_ and *e_g_*, with solid red and dashed blue lines, respectively. The tops of the VBs were set to zero. For *O_h_*-K_2_MnF_6_ and *C*_3*v*_-K_2_MnF_6_, the crystal field splitting (10*Dq*, defined as the differential energy between *t*_2*g*_ and *e_g_* levels) was estimated to be 1.79 and 2.68 eV, respectively. Without accounting for the lattice relaxation effect, the 10*Dq* of *O_h_*-K_2_SiF_6_ and *C*_3*v*_-K_2_SiF_6_ were estimated to be 3.52 and 3.44 eV, respectively. They fell to 2.63 and 2.53 eV when the lattice relation effect was taken into account. In the case of *D*_3*d*_-K_2_GeF_6_ and *C*_3*v*_-K_2_GeF_6_, the 10*Dq* was determined to be 2.76 and 2.72 eV, respectively. After accounting for the lattice relaxation effect, they fell to 2.52 and 2.61 eV, respectively.

### 3.3. Multiplet Energy Levels

Since the d–d transitions of K_2_XF_6_: Mn^4+^ was prohibited by the parity selection rule, the transition probabilities could not be determined. As a result, this report is restricted to energy levels. We estimated the doublet states ^2^E,.^2^T_2,_ and ^2^T_1,_ as well as the quartet states ^4^T_2_ and ^4^T_1a_. The absorption transitions start from the ground ^4^A_2_ state to ^4^T_2_ and ^4^T_1a_ states which often appeared as wide bands and were referred to as the U- and Y-band, respectively. On the other hand, the emission transition started from the ^2^E state to the ground ^4^A_2_ state, which generally appeared as a sharp line and was referred to as R-line.

**Figure 3:**
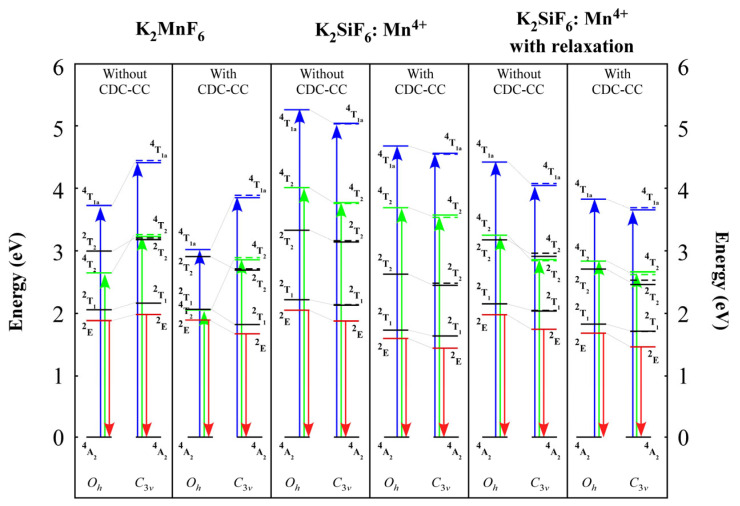
Pure K_2_MnF_6_ and K_2_SiF_6_: Mn^4+^ multiplet energy diagrams. Additionally, demonstrated is the impact of CDC, CC, and lattice relaxation. The left side of each column explains the calculation using *O_h_*-symmetric clusters, while the right side describes the calculation using *C*_3*v*_-symmetric clusters. Black and red lines denote the doublet and quartet states, respectively. When the lower symmetry (*C*_3*v*_) was used, these states were further divided into the *a* (dashed lines) and *e* (solid lines) categories. There are the doublet states ^2^E,.^2^T_2,_ and ^2^T_1,_ as well as the quartet states ^4^T_2_ and ^4^T_1a_. The ^4^A_2_ is the ground state. The absorption occurred during the electronic transitions from the ground ^4^A_2_ state to ^4^T_2_ and ^4^T_1a_ states (U- and Y-band, respectively), as illustrated by the green and blue arrows. The emission, on the other hand, happened as an electronic transition from the ^2^E state to the ground ^4^A_2_ state (R-line), as illustrated by the red arrow. More information can be found in the text. Pure K_2_MnF_6_ and K_2_SiF_6_: Mn^4+^ multiplet energy diagrams. Additionally, demonstrated is the impact of CDC, CC, and lattice relaxation. The left side of each column explains the calculation using *O_h_*-symmetric clusters, while the right side describes the calculation using *C*_3*v*_-symmetric clusters. Black and red lines denote the doublet and quartet states, respectively. When the lower symmetry (*C*_3*v*_) was used, these states were further divided into the *a* (dashed lines) and *e* (solid lines) categories. There are the doublet states ^2^E,.^2^T_2,_ and ^2^T_1,_ as well as the quartet states ^4^T_2_ and ^4^T_1a_. The ^4^A_2_ is the ground state. The absorption occurred during the electronic transitions from the ground ^4^A_2_ state to ^4^T_2_ and ^4^T_1a_ states (U- and Y-band, respectively), as illustrated by the green and blue arrows. The emission, on the other hand, happened as an electronic transition from the ^2^E state to the ground ^4^A_2_ state (R-line), as illustrated by the red arrow. More information can be found in the text.

The pure K_2_MnF_6_ and K_2_SiF_6_: Mn^4+^ computed multiplet energy diagrams with *O_h_* and *C*_3*v*_ symmetry are shown in Figure 3. A few adjustments, including CDC, CC, and lattice relaxation, were also assessed. Figure 3 demonstrates that quite often, the doublet states decreased when reduced symmetry was employed. Furthermore, CDC-CC correction had a smaller impact on *O_h_*-K_2_MnF_6_ than it did on *C*_3*v*_-K_2_MnF_6_, suggesting that *C*_3*v*_-K_2_MnF_6_ benefited more from correlation correction. On the other hand, the quartet states increased for pure K_2_MnF_6_ from *O_h_* to *C*_3*v*_ while they dropped for K_2_SiF_6_: Mn^4+^ in the same order. This was expected because the Mn-F bond length, which varied widely depending on the material, primarily affected the quartet states.

**Figure 4:**
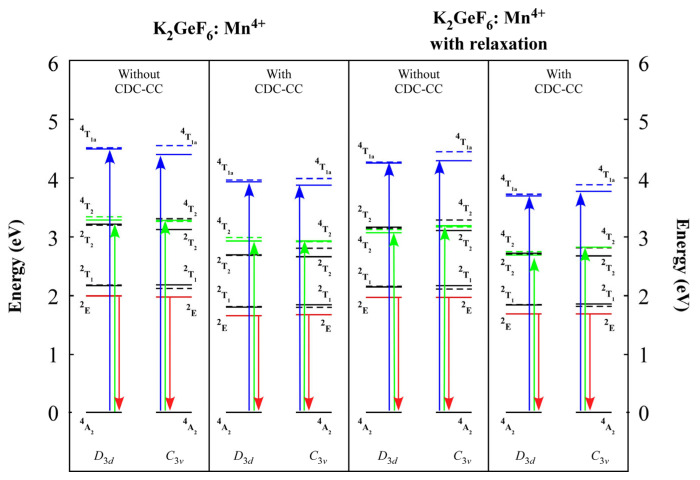
K_2_GeF_6_: Mn^4+^ multiplet energy diagrams. Additionally demonstrated is the impact of corrections, including CDC, CC, and lattice relaxation. A calculation using clusters with *D*_3*d*_ symmetry is described on the left side of each column, while a calculation using clusters with *C*_3*v*_ symmetry is described on the right side. The *O_h_* symmetry notations, in this instance, were borrowed. Black and red lines denote the doublet and quintet states, respectively; dashed (*a* level) and solid lines (*e* level) denote the multiplet splitting. There are the doublet states ^2^E,.^2^T_2,_ and ^2^T_1,_ as well as the quartet states ^4^T_2_ and ^4^T_1a_. The ^4^A_2_ is the ground state. The absorption occurred during the electronic transitions from the ground ^4^A_2_ state to ^4^T_2_ and ^4^T_1a_ states (U- and Y-band, respectively), as illustrated by the green and blue arrows. The emission, on the other hand, happened as an electronic transition from the ^2^E state to the ground ^4^A_2_ state (R-line), as illustrated by the red arrow. More information can be found in the text.

The predicted multiplet energy diagrams of K_2_GeF_6_: Mn^4+^ with *D*_3*d*_ and *C*_3*v*_ symmetry are shown in Figure 4. CDC, CC, and lattice relaxation were also evaluated, similar to Figure 3. These findings showed that the average doublet state values for the two clusters were remarkably similar. Low symmetry was also found to have an impact on multiplet splitting. While the splitting of the ^4^T_2_ state decreased, it increased for the ^4^T_1a_, ^2^T_2,_ and ^2^T_1_ states.

### 3.4. Coulomb Integrals

The Coulomb integrals of pure K_2_MnF_6_, K_2_SiF_6_: Mn^4+^, and K_2_GeF_6_: Mn^4+^ are shown in Table 2. When low symmetry was used, the effective Coulomb integrals $J_{\mathrm{eff}}$ estimated by $c\lambda J_{\mathrm{AO}}$ almost always decreased. Although the $J_{\mathrm{eff}\left(t2g\right)}$ of K_2_GeF_6_: Mn^4+^ without lattice relaxation was greater than that of *D*_3*d*_-K_2_GeF_6_: Mn^4+^, its tendency improved when lattice relaxation was considered. These findings suggest that reduced symmetry resulted in a smaller Coulomb integral. As a result, the decreasing trend of R-line energy could be attributed to a decreased electron–electron repulsion.

## 4. Discussion

LEDs are now used in a variety of commonplace applications, including display backlights for smartphones, tablets, and televisions, as well as warm-white LEDs for energy-efficient lighting. A portion of the blue light from the LED chip is converted into white light by color-converting luminescent materials. This is accomplished by using doped wide-bandgap materials, also referred to as phosphors or Colloidal Quantum Dots (QDs). The color quality of white LEDs is improved when red-emitting phosphors are added when compared to the prototype’s arrangement of a blue LED and a yellow Y_3_Al_5_O_12_: Ce^3+^. These luminous materials have an incredibly high luminescence efficiency, especially at room temperature and above, due to the involvement and stimulation of thermal phonons.

A rare-earth ion such as Eu^2+^ and Ce^3+^ or a transition metal such as Mn^2+^ and Mn^4+^ are doped into an inorganic host material to create phosphor materials. Rare-earth ions are frequently used in conventional LED phosphors, with the main component being Y_3_Al_5_O_12_: Ce^3+^ (YAG: Ce) [39]. By altering the host compound’s composition, the dopant Eu^2+^ can change the emission spectrum, for example, from a green emission in SrGa_2_S_4_: Eu^2+^ [3,4] and SrSi_2_O_2_N_2_: Eu^2+^ [5] to a red emission in Sr_2_Si_5_N_8_: Eu^2+^ [5,6], (Ca, Sr)S: Eu^2+^ [3,7], CaAlSiN_3_: Eu^2+^ [8] and Sr[LiAl_3_N_4_]: Eu^2+^ [9]. A current trend toward creating non-rare-earth element LED phosphors is the result of environmental challenges, including the scarcity of rare-earth materials.

The requirements for a phosphor to be suitable for LED applications were described by Smet et al. [39] in detail. According to the Color Quality Scale (CQS) [40] or the CRI [41], the resulting white light source had a high color rendering. This was significant for illumination. For display applications to produce a broad color spectrum or high color purity, saturated colors were necessary. The lower the filtering losses, the better the phosphors’ emission spectrum fits the color filters. Second, a phosphor must have a high LER, which is a metric for the average eye sensitivity of the spectrum, measured in lm/W) and a high Internal Quantum Efficiency (IQE), which is stable at high temperatures. Third, there needs to be significant blue light absorption, which raises the EQE. A phosphor can only be considered a serious candidate for LED applications when all four requirements are satisfied simultaneously.

The Mn^4+^ emission center prefers to remain in the octahedral or modified octahedral position of the host due to the large ligand-field stabilizing energy of Mn^4+^ in the six-fold coordination. In the initial LFT simulation, only the octahedral (*O_h_*) crystal field was taken into account [42]. The doubly degenerate *e_g_* level had +6*Dq* more energy than the fivefold degenerate 3d level, and the triply degenerate *t*_2*g*_ level had −4*Dq* more energy. The intensity of the crystal field, *Dq*, changed based on the ion-crystal combination, and as a result, the crystal field splitting was 10*Dq*. Through the use of the electron–electron repulsion parameters *A*, *B*, and *C*, also referred to as Racah parameters, the impact of covalency could also be taken into account in this situation. The crystal field strength *Dq*, the Racah parameters B and C, and the multiplet energy levels *E_i_* could thus be used to explain them. The recognized Tanabe Sugano diagrams [21,43], which depict *E_i_*/*B* as functions of *Dq*/*B* for a fixed value of *C*/*B*, describe the energy levels of all d*^N^* systems in an octahedral crystal field as functions of *Dq*. The spectral characteristics of red phosphor materials were also described using the absorption and emission spectra. There were certain doublet states, such as ^2^E, ^2^T_1_, etc., and some quartet states, such as ^4^A_2_, ^4^T_2_, ^4^T_1a_, ^4^T_1b_, etc., since Mn^4+^-doped compounds contained three electrons filling ten degenerate 3d orbitals (3d^3^). The energy was lowest in the ground state (^4^A_2_). The transitions from ^4^A_2_ to ^4^T_2_ (U-band) and ^4^T_1a_ (Y-band) were utilized for absorption, while the transition from ^2^E to ^4^A_2_ (R-line) was employed for emission when used as red phosphor materials.

Previously, we studied the potential of oxide and fluoride materials for red phosphor materials in WLED by DV-Xα and DVME methods. The investigation included lattice relaxation, orbital energy, multiplet energy, absorption spectra, energy correction, pressure dependence, and emitted light utilizing CIE 1931 color space [35,44,45,46,47,48,49,50,51,52]. Because of the length of the Mn-F bond, the quartet state energies typically had a strong relationship with crystal field splitting. On the other hand, doublet state energies strongly depended on the correlation correction. The computational conditions to reproduce those optical properties depend on the material itself.

The study of low-structure symmetrization was required when understanding the properties of novel phosphor materials. According to our findings, a low structure had a substantial effect on multiplet structures, which could affect their performance as phosphor materials. Table 1 indicates that considering the lattice relaxation effect caused by the Mn^4+^, substitution resulted in a considerable shift in the bond lengths. The crystal field splittings were approximated using the one-electron DV-Xα approach, as shown in Figure 2. The 10*Dq* crystal field splitting tendency was caused by the lengths of the Mn-F bonds. Furthermore, the multiplet energies in Figure 3 and Figure 4 were determined using the many-electron DVME approach. The splitting of the corresponding multiplet energy levels was visible in lower structure symmetrization.

## 5. Conclusions

By taking into consideration lattice relaxation, CDC, and CC, the multiplet energies of Mn^4+^ doped K_2_XF_6_ crystals were qualitatively estimated. The fluoride compounds exhibited here were suitable materials to be used as red phosphors for white LEDs since the Mn^4+^ impurities in these hosts were emitted at approximately 620−630 nm (the proper spectral range to obtain a “warm” white light from the LEDs). In addition, they appeared to be thermally stable since they are known as commercial red phosphors (especially K_2_SiF_6_: Mn^4+^). We found that the Mn-F bond length dropped, yet the U- and Y-band energies increased. By contrasting various fluoride crystal symmetry polytypes, the impact of lower symmetry was explored. The Mn-F bond length decreased, while the absorption energies of ^4^*A*_2*g*_→^4^*T*_2*g*_ (^4^F) and ^4^*A*_2*g*_→^4^*T*_1*g*_ (^4^F) increased, yet the ^2^*E_g_* → ^4^*A*_2*g*_ emission energy decreased. A reduced Coulomb integral result was produced when symmetry was low. It followed that a decrease in electron–electron repulsion was the cause of the declining trend in R-line energy. The CC factor *c* dominated the R-line energy, while the Mn-F bond length and crystal field splitting was principally responsible for the U- and Y-band energies. We discovered that low symmetry decreased the attraction between the electrons.

## Figures and Tables

**Table 1:** Mn-F Bond lengths (Å) of *O_h_*-K_2_MnF_6_, *C*_3*v*_-K_2_MnF_6_, *O_h_*-K_2_SiF_6_, *C*_3*v*_-K_2_SiF_6_, *D*_3*d*_-K_2_GeF_6_, and *C*_3*v*_-K_2_GeF_6_ doped with Mn^4+^.

K_2_XF_6_ Crystals	*d*1	*d*2	*d*3	*d*4	*d*5	*d*6
Without relaxation						
*O_h_*-K_2_MnF_6_	2.005920	2.005920	2.005920	2.005920	2.005920	2.005920
*C*_3*v*_-K_2_MnF_6_	1.785311	1.785311	1.785314	1.792269	1.792271	1.792271
*O_h_*-K_2_SiF_6_: Mn^4+^	1.682920	1.682920	1.682920	1.682920	1.682920	1.682920
*C*_3*v*_-K_2_SiF_6_: Mn^4+^	1.680538	1.680542	1.680542	1.688463	1.688463	1.688461
*D*_3*d*_-K_2_GeF_6_: Mn^4+^	1.770284	1.770280	1.770280	1.770284	1.770284	1.770284
*C*_3*v*_-K_2_GeF_6_: Mn^4+^	1.777360	1.777357	1.777357	1.805570	1.805570	1.805564
With relaxation						
*O_h_*-K_2_MnF_6_	2.005920	2.005920	2.005920	2.005920	2.005920	2.005920
*C*_3*v*_-K_2_MnF_6_	1.785311	1.785311	1.785314	1.792269	1.792271	1.792271
*O_h_*-K_2_SiF_6_: Mn^4+^	1.807000	1.807000	1.807000	1.807000	1.807000	1.807000
*C*_3*v*_-K_2_SiF_6_: Mn^4+^	1.802744	1.802753	1.802753	1.811248	1.811248	1.811247
*D*_3*d*_-K_2_GeF_6_: Mn^4+^	1.810000	1.809997	1.809997	1.810000	1.810000	1.810000
*C*_3*v*_-K_2_GeF_6_: Mn^4+^	1.795749	1.795750	1.795750	1.824250	1.824250	1.824244

**Table 2:** Using the MnF_6_^2−^ model clusters with *O_h_*, *D*_3*d*_, and *C*_3*v*_ symmetry, Coulomb integral (eV) for the pure TM−3d atomic orbitals ($J_{\mathrm{AO}}$) and the molecular orbitals ($J_{\mathrm{MO}}$) were calculated. The adjustments were contrasted, including those with and without lattice relaxation. The orbital deformation parameter (λ) and the correlation correction factor (*c*) were multiplied by $J_{\mathrm{AO}}$ to calculate the effective Coulomb integrals ($J_{\mathrm{eff}}$).

Compound	K_2_MnF_6_		K_2_MnF_6_: Mn^4+^		K_2_SiF_6_: Mn^4+^ Relaxed		K_2_GeF_6_: Mn^4+^		K_2_GeF_6_: Mn^4+^ Relaxed	
Symmetry	*O_h_*	*C*_3*v*_	*O_h_*	*C*_3*v*_	*O_h_*	*C*_3*v*_	*D*_3*d*_	*C*_3*v*_	*D*_3*d*_	*C*_3*v*_
$J_{\mathrm{AO}}$	23.92	24.30	24.36	24.12	24.21	23.88	24.35	24.29	24.31	24.26
$J_{\mathrm{MO}\left(t2g\right)}$	19.19	19.96	20.40	19.30	19.91	18.35	20.07	19.95	19.91	19.88
$J_{\mathrm{MO}\left(eg\right)}$	16.50	18.43	19.08	18.91	18.09	17.93	18.66	18.32	18.38	18.18
$\lambda_{\left(t2g\right)}$	0.80	0.82	0.84	0.80	0.82	0.77	0.82	0.82	0.82	0.82
$\lambda_{\left(eg\right)}$	0.69	0.76	0.78	0.78	0.75	0.75	0.77	0.75	0.76	0.75
*c* factor	1.01	0.84	0.78	0.77	0.85	0.84	0.83	0.85	0.86	0.86
$c\lambda_{\left(t2g\right)}$	0.81	0.69	0.65	0.61	0.70	0.64	0.69	0.69	0.70	0.70
$c\lambda_{\left(t2g\right)}$	0.70	0.64	0.61	0.60	0.64	0.63	0.64	0.64	0.65	0.64
$J_{\mathrm{eff}\left(t2g\right)}$	19.42	16.81	15.86	14.77	16.94	15.39	16.69	16.87	17.07	17.05
$J_{\mathrm{eff}\left(eg\right)}$	16.70	15.52	14.83	14.47	15.39	15.04	15.51	15.49	15.75	15.60

## Data Availability

No new data were created.
